# Optimization of an *in Silico* Protocol Using Probe Permeabilities to Identify Membrane Pan-Assay Interference Compounds

**DOI:** 10.1021/acs.jcim.2c00372

**Published:** 2022-06-13

**Authors:** Pedro R. Magalhães, Pedro B. P. S. Reis, Diogo Vila-Viçosa, Miguel Machuqueiro, Bruno L. Victor

**Affiliations:** † BioISI - Biosystems & Integrative Sciences Institute, Faculty of Sciences, University of Lisboa, Campo Grande, C8 bdg, 1749-016 Lisboa, Portugal

## Abstract

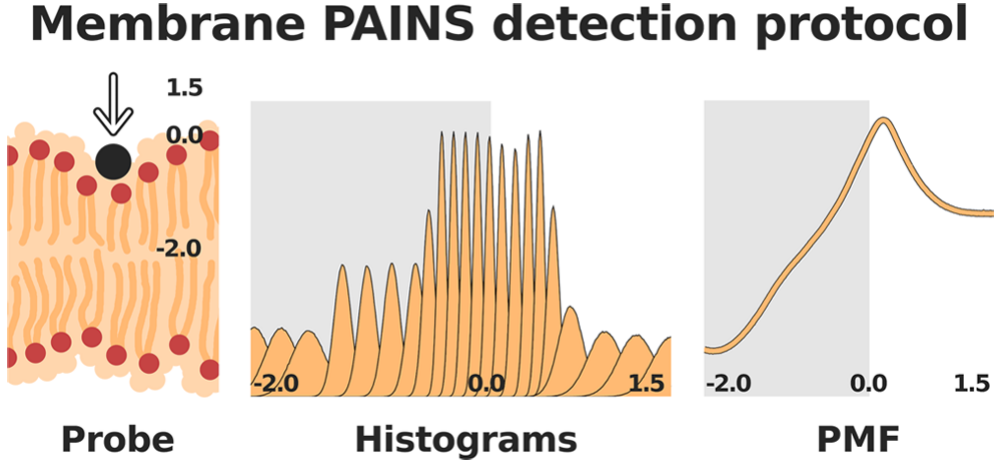

Membrane pan-assay interference compounds (PAINS) are a class of molecules that interact nonspecifically with lipid bilayers and alter their physicochemical properties. An early identification of these compounds avoids chasing false leads and the needless waste of time and resources in drug discovery campaigns. In this work, we optimized an *in silico* protocol on the basis of umbrella sampling (US)/molecular dynamics (MD) simulations to discriminate between compounds with different membrane PAINS behavior. We showed that the method is quite sensitive to membrane thickness fluctuations, which was mitigated by changing the US reference position to the phosphate atoms of the closest interacting monolayer. The computational efficiency was improved further by decreasing the number of umbrellas and adjusting their strength and position in our US scheme. The inhomogeneous solubility-diffusion model (ISDM) used to calculate the membrane permeability coefficients confirmed that resveratrol and curcumin have distinct membrane PAINS characteristics and indicated a misclassification of nothofagin in a previous work. Overall, we have presented here a promising *in silico* protocol that can be adopted as a future reference method to identify membrane PAINS.

## Introduction

High-throughput screening is a commonly used approach in drug discovery campaigns to identify compounds showing activity to a specific therapeutic target.^1^ Depending on the used test readout, certain molecules can emerge as hits without actually interacting with the desired target. In addition to this lack of specificity, such compounds can also be promiscuous and show activity in different independent assays.^2−4^ Such “frequent hitters”, commonly known as false-positives, are impossible to optimize and consequently do not lead to a successful drug development process, wasting time and resources. Therefore, the ability to identify such compounds in the early steps of drug discovery campaigns is mandatory for small-to-large pharma and biotech companies.^4^

This class of promiscuous compounds was named in 2010 by Baell and Holloway^2^ as pan-assay interference compounds (PAINS) and gained more attention in this past decade.^4^ PAINS comprise a large variety of compounds with different sources of diverse behavior or assay interference. Compound fluorescence events,^5^ chelation,^6^ chemical aggregation,^7^ redox activity,^8^ membrane perturbation/disruption,^9^ and nonselective compounds^10^ are just a few examples of characteristic interference chemicals. Although the selectivity problems associated with PAINS have been the main focus of the scientific community, there are other categories, like phytochemicals, that, due to the compound large abundance and perceived health benefits, gained a lot of recent attention.^11^ Phytochemicals are one of the major components of plants and have long been used in traditional medicine to treat several different health problems.^12^ The molecular mechanism associated with these compounds has been conventionally interpreted or theorized by effects on receptors, biological pathways, ion channels, and transporters.^13^ However, their broad pharmacological activity spectra make it unfeasible for these compounds to target any specific protein. Furthermore, their activity modulation of apparently unrelated proteins led different authors to pinpoint the interaction of these compounds with cell membranes as the underlying mechanism behind their promiscuity.^9,13^ These special phytochemicals are currently known as membrane PAINS since they affect membrane physicochemical properties, such as curvature, fluidity, viscosity, elasticity, and permeability.^14,15^ These membrane perturbations are more pronounced than common protein/membrane-binding phenomena and seem to have more points of contact with membrane-acting drugs, such as anesthetic, cholinergic, anti-inflammatory, adrenergic, and antitumor compounds.^13^

In recent years, there has been a large interest in the development of computational methods to identify membrane PAINS and characterize their mode of action.^9,16,17^ The developed methodologies focused on quantifying the membrane deformations due to the presence of embedded potential membrane PAINS. Ingólfsson and co-workers^9^ explored the membrane perturbation effects of several phytochemicals in membranes and mechanosensitive membrane proteins through a combination of gramicidin-based assays (experimental) and coarse-grained molecular dynamics (MD) simulations. The developed protocol, which relied on computationally demanding umbrella sampling (US) simulations, worked as the basis for several subsequent methodologies.^16−18^

The new implementations of the US protocol increased the molecular detail using atomistic MD and significantly improved the description of the membrane energy barriers.^9,16,17^ However, such methodologies are very computationally intensive, leading to limitations in their implementation, such as the use of single replicates and short MD simulations.^16,17^ In this work, we have implemented a new optimized computational protocol for the identification and characterization of membrane PAINS. Along the same lines as previous implementations, our approach uses atomistic MD simulations coupled to a US scheme to calculate the potential of mean force (PMF) energy profiles. It uses a Lennard-Jones probe to evaluate the effects of different compounds with varying degrees of reported membrane PAINS character, namely, curcumin, resveratrol, and nothofagin.^9,16^ We have tested the use of long MD simulations, multiple replicates, different US schemes, and different atoms as US reference groups.

## Methods

### System Setup and MM/MD Parameters

We started from a pre-equilibrated lipid bilayer system consisting of 128 1-palmitoyl-2-oleoyl-*sn*-glycero-3-phosphocholine (POPC) lipids solvated by ∼6000 water molecules.^16^ This was also used as the template to build all membrane PAINS systems by adding curcumin (CUR), nothofagin (NOT), or resveratrol (RES) molecules. The compounds were evenly and randomly distributed between membrane leaflets and POPC in a 1:10 molar ratio (similarly to Ingólfsson et al.^9^), resulting in different starting systems: pure POPC, POPC+CUR, POPC+NOT, and POPC+RES. An additional system containing a 2:10 molar ratio of POPC to CUR molecules (CUR24) was also built following the same protocol.

Molecular dynamics simulations were performed using GROMACS 2018.6^19−21^ and the united-atom GROMOS 54A7 force field.^22^ Topologies for the compounds were obtained using the automated topology builder server (ATB),^23−25^ as previously described.^16,18^ The force field parameters used for POPC were the ones included in GROMOS 54A7.^26,27^

Long-range electrostatic interactions were computed with the particle mesh Ewald (PME) method^28,29^ using a Fourier grid spacing of 0.12 nm and a cutoff of 0.9 nm for direct contributions. Lennard-Jones interactions were calculated using a nonbonded neighbor pair list with a cutoff of 0.9 nm, allowing the use of a cutoff scheme.^30^ Lipid and PAINS bonds were constrained with the parallel linear constraint solver (P-LINCS),^31^ while water molecules were constrained using the SETTLE algorithm.^32^ The simple-point charge (SPC) water model was used.^33^

The system was coupled to a temperature bath at 298.15 K using the v-rescale thermostat^34^ with a coupling constant of 0.1 ps. A semi-isotropic Parrinello–Rahman barostat^35,36^ was used in order to keep a constant pressure of 1 bar with a coupling constant of 2.0 ps and a compressibility of 4.5 × 10^–5^ bar^–1^.

The energy of each system was minimized using the steepest descent algorithm^37^ in two steps: first, with no constraints and with a maximum step size of 0.0001 nm; second, with all bonds constrained and a maximum step size of 0.001 nm. The tolerance was set to 0.0 kJ mol^–1^ nm^–1^ in both steps, meaning the algorithm stopped when reaching machine precision. The velocities for each system were then generated according to a Maxwell distribution at 298.15 K varying the initial seed. These system initializations were performed for 200 ps with a time step of 2 fs using the MD integrator.

Systems were pre-equilibrated in 200 ns-long unbiased MD simulations in order to assess how the presence of PAINS affected membrane bulk properties, such as the total *x*/*y* area (Figure S1). The systems were considered to be equilibrated after 100 ns. Using the final converged 100 ns of the unbiased MD simulations, we calculated the average insertion of the different PAINS compounds in the bilayer (Figure S2). The insertion relative to the near phosphate monolayer was calculated using the geometric center of each PAINS compound. The preferred insertion regions for each compound are also illustrated in the snapshots shown in the right panel of Figure S2.

### Umbrella Sampling

To prepare each system for the umbrella sampling simulations, we added a probe defined as a Lennard-Jones “sphere” with an overall size comparable to a benzene molecule, as described in ref (16). The main role of the probe particle is to detect membrane perturbations. If the probe is too small, it will lose sensitivity, while if it is too large, it may itself perturb the membrane. Therefore, all probe sizes between these two extremes should lead to similar results. Different replicates were then built by replacing one of the bulk water molecules in each system at random. We ran a steered MD simulation for each system in which the probe is gradually pulled in the *z* coordinate across the membrane normal (with a force of 1000 kJ mol^–1^ nm^–2^ and a velocity of 1 nm/ns) while keeping the *xy* coordinates restrained. From these simulations, we selected several initial conformations with the probe placed from the center of the bilayer to the bulk water every 0.1 nm. The force constant (*K*_f_) used in these umbrellas was 1000 kJ mol^–1^ nm^–2^. For each of these initial conformations, we performed 600 ns-long umbrella sampling simulations with the initial 300 ns being discarded for equilibration. This conservative approach was based on several structural properties, such as the local monolayer thickness and deformation, which proved harder to converge in the umbrellas near the membrane/water interface (Figure S3). According to our findings, the use of 300 ns of equilibration assured that any initial conformational bias was removed or, at least, well-mitigated. Since the most difficult part to converge is the phosphate region, only after applying such a long protocol, we managed to decrease the dispersion between replicates. In addition to the general protocol described above, some variations in the number and positions of the umbrellas (as well as their corresponding *K*_f_ values) were introduced. The details of these variations are included and discussed in the Results and Discussion.

### Analyses and Error Calculations

The potential of mean force (PMF) profiles for each system were calculated using the weighted-histogram analysis method (WHAM)^38^ implemented in GROMACS. Membrane permeabilities were calculated using the inhomogeneous solubility-diffusion model (ISDM),^39,40^ implemented in a software package developed by Vila-Viçosa and co-workers^41^ based on the formalism described in refs (42 and 43) and succinctly described in the Supporting Information. The position-dependent diffusion values of the probe are computed using the autocorrelation function of its *zz*-position at each umbrella window. Combining the diffusion and the PMF values, we can calculate the position-dependent resistance profile that can then be integrated to obtain the overall permeation coefficient (shown in Figure S4). The standard error values included in the figures and tables were obtained using a modified jackknife resampling approach. This method uses combinations of replicates of a given system in a leave-one-out strategy. Thus, using *n* combinations of *n* – 1 replicate subsamples, we can estimate the standard error values of the original sampling.^44^ This approach has the advantage of avoiding the error estimation of system properties from single replicate averages that may have convergence issues.

The local membrane thickness was obtained by calculating the half thickness for each monolayer using all P atoms within a radius cutoff (10 Å) in the *xy* plane centered on the probe, while the membrane center is calculated using all P atoms outside of a secondary 15 Å radius, i.e., the P atoms that are unperturbed by the probe.^45,46^ These calculations were performed using the *MembIT* tool (https://github.com/mms-fcul/MembIT).

Other analyses, were performed using either GROMACS or in-house tools with all plots and figures being generated using Gnuplot,^47^ PyMOL,^48^ and GIMP.^49^

## Results and Discussion

### Protocol Optimization

The umbrella sampling biases over the course of an entire 600 ns-long simulation of replicate 1 of a pure POPC system are shown in Figure S5A. This system, which we designate as 37U^B^, contains 37 umbrellas, each spaced 0.1 nm, and uses the bilayer center as the reference; i.e., the umbrella at 0.0 is located in the center of the bilayer, while the umbrella at 3.6 is in the bulk water. As previously mentioned (see the Methods), the umbrellas located near the membrane/water interface are the most perturbed and, thus, more difficult to converge (around 1.9 nm), while the ones located the furthest away from this interface are the least perturbed. It should be noted that discarding the initial 300 ns of these simulations does not seem to affect the proper sampling of the different umbrellas. When the equilibrated regions of all replicates are taken into account, we obtain the population histogram shown in Figure S5B. Despite the observed small difficulty in the aforementioned membrane/water interface region, there is a high and consistent overlap between all neighboring umbrellas, which guarantees that a good sampling of the simulated system is achieved.

**Figure 1:**
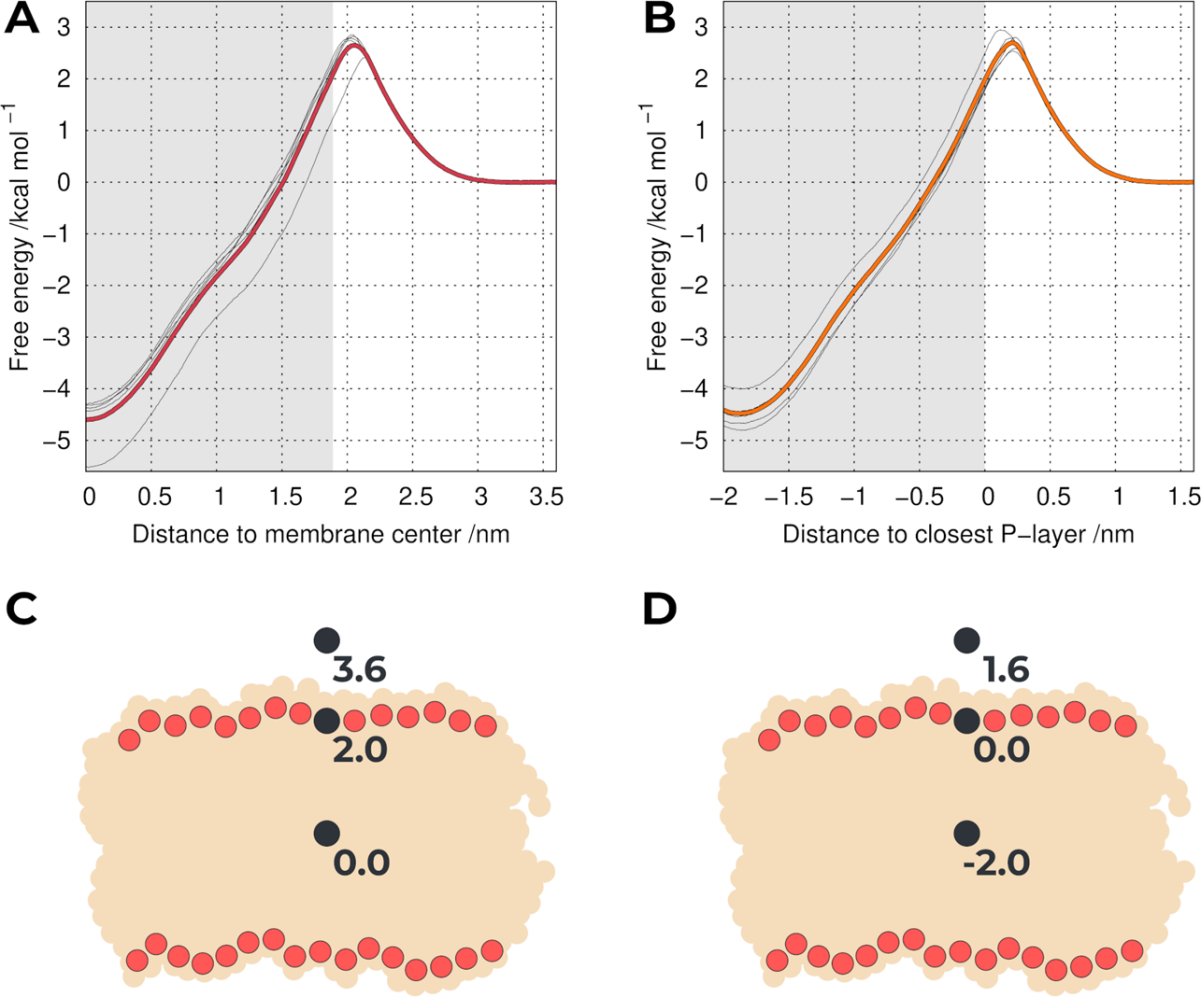
PMF of translocating a probe across a POPC bilayer using either the center of the membrane as the reference (A) or the closest P-layer (B). The thicker colored lines include all replicates, whereas the thinner gray lines correspond to individual replicates. The gray area is half the average bilayer thickness. Cartoons of the relative positions of the probe in the *z*-axis using the membrane as a reference (C) or the closest P-layer (D).

We computed the PMF profiles using the entire sampling for the 37U^B^ system (Figure S5B) or by separating the individual replicates (Figure 1A). Although the system is composed only of the pure POPC bilayer and the probe, there is substantial variability between some replicates. This illustrates the importance of using long equilibration times with several replicates, since using a shorter single replicate^16^ can result in an underestimated or overestimated free energy. As previously remarked,^50^ these differences are the consequence of the heterogeneity in the membrane; i.e. in different replicates, the probe can cross the headgroup layer of the membrane in different regions with different compactness, thus encountering different resistances. We observed a clear similarity between our PMF profile (Figure 1A) and the one obtained by Jesus et al.,^16^ in particular, the relative location of the energy maximum and the overall shape. These shared characteristics cannot be dissociated from the fact that they shared the same probe parameters and lipid force field. However, these PMF profiles differ significantly from the one published by Ingólfsson et al.^9^ As previously discussed,^16^ the extra detail in the atomistic (united-atom) simulations, compared with coarse-grained MARTINI, seems to be very important to discriminate compounds with different membrane PAINS characteristics. The major differences observed between the resulting PMF profiles are found at (i) the center of the lipid bilayer with MARTINI creating an artificial energy barrier, which is corrected in the atomistic simulations; (ii) at the phosphate region, where the coarse-grained force field does not correctly model the expected energy barrier of a hydrophobic probe. This limitation is particularly important since the changes in the phosphate group region seem to be more impactful for membrane structural stability and permeability.

As observed in the PMF profile (Figure 1A), the main source of variability between replicates occurs at the peak of the energy barrier, which corresponds to the region where the probe encounters the most resistance, i.e., at the P-headgroup region.

Depending on the simulated replicate, the probe interacts with heterogeneous P-headgroup packing environments with different membrane thickness fluctuations, which can take hundreds of nanoseconds to equilibrate. The observed differences are also likely related to the initial definition of the membrane center, which was calculated by the average position along the membrane normal of all P atoms. In theory, the choice of the reference point should have no effect on the final PMF. However, in most systems, this is not true since there are always sampling limitations. In an attempt to reduce some of the variability between replicates, we used as a new US reference the average position of the closest monolayer P atoms. This new reference axis becomes analogous to a measure of the probe monolayer insertion, where the new 0.0 nm umbrella corresponds to the average position of the closest P atoms, while an umbrella at −2.0 nm corresponds to a deeper insertion, near the membrane center. This new system, which we termed 37U^M^ (Figure 1B), still does not account for all local deformations in the membrane, but most of the larger membrane thickness fluctuations are attenuated, reducing the impact of some individual outlier replicates. It is also worth noting that this change in the US reference did not impact the quality of the sampling (Figure S6). Although this is just a simple change in the reference position, it already leads to a better description of membrane perturbation by the probe. In fact, this will be particularly important to deal with increased system complexity, as in those in the presence of potential PAINS compounds.

We still observe some remaining heterogeneity present in the 37U^M^ system, which is evidenced in the peak of the energy barrier (Figure 1B). This is mainly due to local membrane deformation events, which are also difficult to equilibrate between replicates in our time scale. In an attempt to quantify this local deformation phenomenon and understand how the first coordination sphere of the P atoms interacts with the probe, we calculated the local monolayer thickness (Figure S7). When the probe is located outside and near the P-headgroup region, its presence creates a local depression; i.e., the phosphate atoms are pushed down toward the center of the bilayer, reducing the monolayer thickness. In contrast, when the probe is right below the P-headgroup region, the opposite effect is observed, as the phosphate groups cover the probe, creating a protrusion and increasing the value of the thickness.

After significantly attenuating the membrane heterogeneity in the probe insertion reference, we need to decrease the computational load of using 5 replicates and 37 umbrellas along the membrane insertion pathway. We identified three approaches to accomplish this: reduce the number of replicates, reduce the length of simulations, or reduce the number of individual umbrellas. All these approaches would lead to the desired effect; however, the first two are more likely to result in sampling issues. A decrease in the number of replicates would inevitably reduce our sensitivity in the error estimations, which could lead to serious limitations when trying to distinguish between systems containing different membrane-perturbing compounds. A reduction in the length of the simulations also seems to be a precarious solution, especially since we need significantly long equilibration runs to eliminate all initial bias introduced in the probe/membrane setup (see the Methods), a limitation that can be worse with the introduction of potential PAINS compounds.

We focused on the reduction in the number of individual umbrellas and, for that, we divided the probe insertion pathway into different regions according to the sampling difficulty. An estimation of this difficulty can easily be inferred from the data already presented (Figure S5) and can be roughly correlated to the proximity to the membrane/water interface. In the regions around the phosphate groups, the sampling was harder and we kept all 0.1 nm-spaced umbrellas, whereas in the easier regions away from the phosphate regions, we managed to increase this spacing distance with a concomitant adjustment of the force constant values (*K*_f_). After some trial-and-error, we decreased the number of umbrella windows to 22 (22U^M^), representing a substantial decrease in the overall computational cost. The number and position of the umbrella windows can not be dissociated from the *K*_f_ values used, and we increased or decreased these values in regions near the phosphate or away from the phosphate groups, respectively (Table S1).

**Figure 2:**
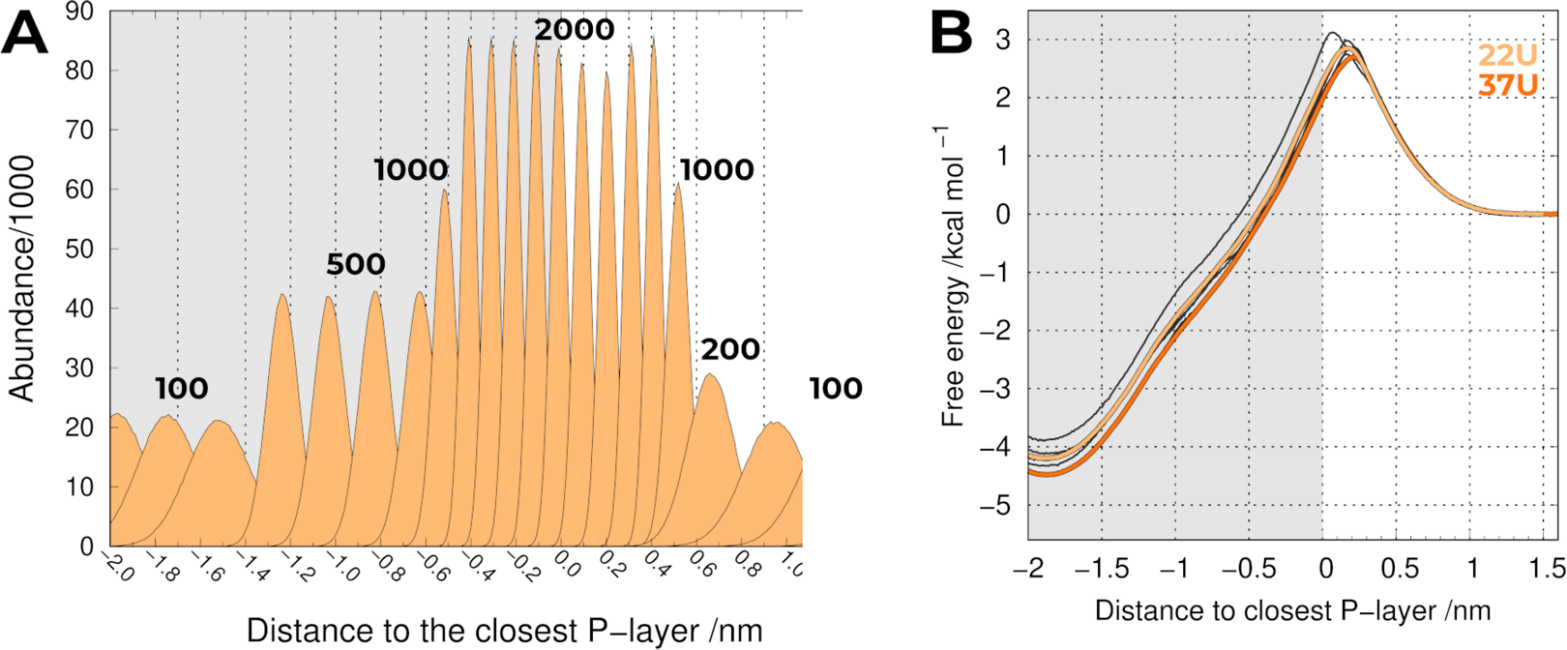
(A) Population histograms for POPC 22U^M^ including all replicates and the corresponding *K*_f_ values (shown in kJ mol^–1^ nm^–2^). (B) PMF of translocating a probe across a POPC bilayer using the closest P-layer as reference using either 22 umbrellas (yellow) or 37 umbrellas (orange). The thicker lines include all replicates, whereas the thinner ones correspond to individual replicates in the 22U^M^ system.

Using this protocol, we managed to maintain the quality of the sampling (overlap between the neighboring umbrellas, Figure 2A).

As shown in Table S1 and Figure 2A, we increased the *K*_f_ value in the region near the P-layer (i.e., −0.4 to 0.4) from 1000 to 2000 kJ mol^–1^ nm^–2^, which was pivotal in attenuating some of the sampling heterogeneity observed in that region. As a result, we obtained more homogeneous PMF profiles (Figure 2B) that very closely resemble the one obtained using 37 umbrellas. Despite our efforts in tightening the constraints in those key umbrellas, some variability can still be observed between replicates, probably due to the inherent differences between the regions where the probe inserts in the membrane (local deformations), which seem very difficult to mitigate in our time scale. This reinforces our decision to keep using five replicates and not to reduce the simulation length. Notwithstanding, there was a significant computational gain from reducing the overall number of umbrellas from 37 to 22. Since the PMF profiles resulting from the three different US protocols used in this work (Figures 1 and 2) are very similar, we will focus on 22U^M^ and take advantage of its reduced computational cost.

The structure of the PMF profiles carries information on the physical state of the membrane and is usually very well-correlated with several other membrane properties.^16,43,51^ Nevertheless, it is not obvious how to assign deviation in specific parts of the profile with the membrane stability and possible deformation due to the presence of different membrane PAINS. Previously, this has been done by looking at the entry/exit energy barriers (size and position)^16^ and at the eventual barrier at the center of the membrane when present.^9^ However, the PMF profile also conveys information on the probe diffusion across the membrane, the resistance encountered and, ultimately, a membrane permeability coefficient.^39−43^ This coefficient has many important membrane properties convoluted in one value and can be particularly advantageous when comparing between membrane systems with different compounds embedded. The permeability coefficient values are calculated using the ISDM method,^39,40^ as previously described.^41−43^ Although ISDM has been successfully used to estimate the membrane permeability to hydrophobic compounds,^41,43^ the method is highly sensitive to the convergence and overall sampling quality of the PMF profiles. However, in our system setup, since we use a hydrophobic sphere as a probe instead of an explicit hydrophobic molecule, the lack of rotational entropy might result in a better convergence.

We calculated the permeability coefficients for the three US protocols used in this work (37U^B^, 37U^M^, and 22U^M^) and observed only small differences between them, all within the error margin (Table 1).The permeability coefficient values seem to be correlated with the entry and exit energy barriers, which are also very similar between the three systems (Table 1). The quality of the PMF profiles obtained for the 22U^M^ system is also expressed in the smaller error observed for this setup. Using this protocol, we increased the complexity of our systems by adding compounds with different degrees of PAINS-like behavior, as described in the literature.^9,16^

**Table 1:** Membrane Permeability Coefficients (cm s^–1^) and Energy Barriers of Entering and Exiting the POPC Bilayer (kcal mol^–1^)^a^

system	permeability	Δ*G*_entry_	Δ*G*_exit_
37U^B^	4.6 ± 0.8	2.6 ± 0.1	7.2 ± 0.1
37U^M^	4.3 ± 0.5	2.7 ± 0.1	7.2 ± 0.1
22U^M^	4.4 ± 0.4	2.9 ± 0.0	7.1 ± 0.1

a The entry barrier (Δ*G*_entry_) is the energy difference between the maximum value (at the membrane/water interface) and bulk water, while the exit barrier (Δ*G*_exit_) is the difference between the global minimum at the membrane center and the previous maximum. Errors were calculated using a jackknife approach.

### Protocol Application to Identify PAINS Compounds

**Figure 3:**
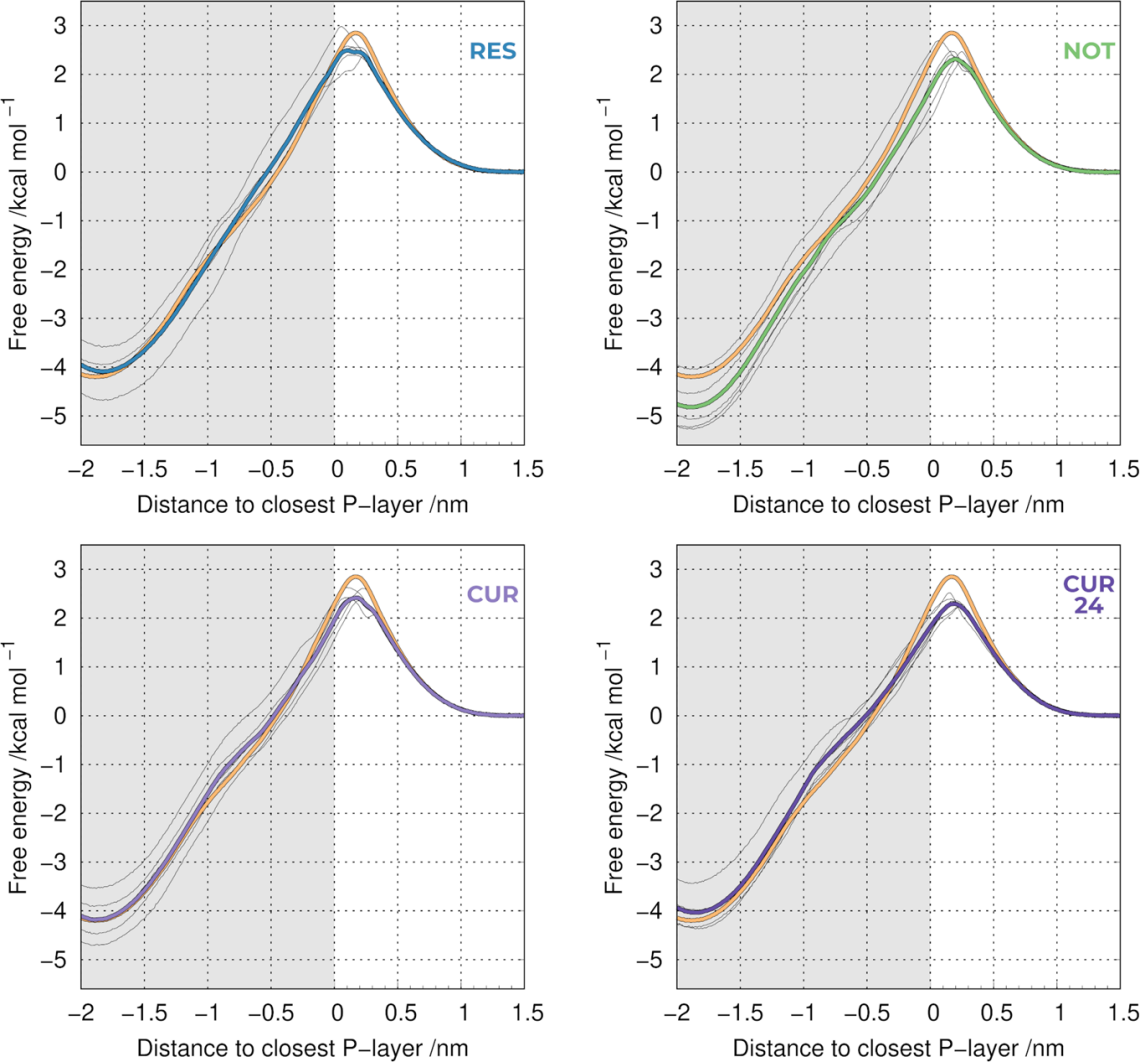
PMF profiles of translocating a probe across a POPC bilayer in the absence or presence of the tested compounds: resveratrol (RES), nothofagin (NOT), curcumin 10% (CUR), and curcumin 20% (CUR24). The thicker lines include all replicates (average), whereas the thinner ones correspond to individual replicates of the system with PAINS compounds. The orange profile corresponds to the compound-free control.

In the previous section, we showed that the 22U^M^ approach was the least computationally demanding option while still retaining the sampling quality and a high degree of homogeneity in the harder-to-sample regions of the membrane. The obtained well-converged PMF profiles allowed us to acquire permeability coefficient values with relatively small errors. We applied this protocol setup to several compounds, known to have different degrees of PAINS-like behavior, as described in the literature:^9,16^ resveratrol (RES), as a mild membrane PAINS, nothofagin (NOT) as a non-PAINS, and curcumin (CUR) as a strong membrane PAINS. The different complex systems were built by embedding each of the three PAINS compounds in the lipid bilayer in a 1:10 mol/mol ratio, evenly distributed between the two leaflets, and then pre-equilibrated using a relatively short unbiased MD simulation, as detailed in the methodology section. An additional system was also prepared using a 2:10 mol/mol ratio for curcumin (termed CUR24), as discussed below. All compounds had an impact on the final PMF profiles obtained when compared to the pure POPC membrane (Figure 3). In all cases, we observe significantly more variability between individual replicates than what was previously observed in the pure POPC systems (Figure 2B). The source for this variability is also the local membrane deformation triggered by the probe insertion in the water/membrane interface. However, the overall heterogeneity is now also impacted by the presence of the compounds, which are not uniformly distributed (Figure S3).

We observe that all compounds change the PMF profile; however, the differences between the compounds are not easily clarified by visual inspection. Furthermore, it is difficult to evaluate how the specific PMF differences affect the membrane properties. To tackle this issue, we calculated the membrane permeability coefficients (Table 2), which provide a quantification of the membrane perturbation and overall stability.

**Table 2:** Energy Barriers of Entry (Δ*G*_entry_) and Exiting (Δ*G*_exit_) the POPC Bilayer and Membrane Permeabilities^a^

system	permeability	Δ*G*_entry_	Δ*G*_exit_
POPC	4.4 ± 0.4	2.9 ± 0.1	7.1 ± 0.1
RES	4.5 ± 0.3	2.5 ± 0.1	6.6 ± 0.1
NOT	5.6 ± 0.6	2.3 ± 0.0	7.1 ± 0.2
CUR	5.0 ± 0.5	2.4 ± 0.0	6.6 ± 0.2
CUR24	5.7 ± 0.3	2.3 ± 0.0	6.3 ± 0.1

a Energy values are shown in kcal mol^–1^, and permeabilities are shown in cm s^–1^. Errors were calculated using a leave-one-out (jackknife) approach.

From the permeability coefficients, resveratrol showed the lowest perturbation to the pure POPC membrane (4.5 ± 0.3 vs 4.4 ± 0.4), which is in agreement with the previously reported description of resveratrol as a mild membrane PAINS.^9^ On the other hand, nothofagin has a more noticeable impact on the PMF (Figure 3), leading to a significant difference in the calculated permeability coefficient (5.6 ± 0.6). This is in disagreement with previous observations that nothofagin is not a membrane PAINS.^16^ This discrepancy is most likely related to the lack of sampling in the work by the Jesus and co-workers,^16^ which used a single 50 ns replicate. In our work, we performed (5×) 300 ns of pre-equilibration MD simulations, followed by 300 ns production MD, to allow convergence in the nothofagin/membrane configuration space. This will mitigate any initial system building bias and allow the correct equilibration of our system properties, such as the local membrane thickness and total area of the membrane patch. As we found in the previous section, both the length and the number of replicates for these simulations are key factors to achieve a good convergence. From our data, it is clear that nothofagin can, in fact, exhibit some membrane PAINS behavior.

We have also used curcumin in our study, which has been identified as a strong membrane PAINS.^9^ We did observe noticeable deviations in the overall PMF profile (Figure 3), leading to an apparent increase in the membrane permeability coefficient value (Table 2). However, the difference of its permeability coefficient, when compared with pure POPC, is within the error values (5.0 ± 0.5 vs 4.4 ± 0.4), which are significant due to the system heterogeneity among individual replicates. Since curcumin has been branded as a strong membrane PAINS,^9^ we expected a more significant membrane perturbation effect. This prompted us to design an extra system (CUR24) with twice the concentration of the compound (2:10 mol/mol ratio). The effect of doubling the concentration of curcumin appeared to be 2-fold: first, an increase in the perturbation effect when compared to pure POPC, as evidenced by the shape of the PMF profile (Figure 3) and the corresponding permeability coefficient (5.7 ± 0.3 vs 4.4 ± 0.4); second, a reduction in the heterogeneity between individual replicates (Figure 3), which is reflected in the smaller error value when compared to several other systems. Overall, it seems that the addition of more curcumin molecules to the lipid bilayer does lead to a statistically significant perturbation effect, hence confirming its membrane PAINS character.

To complement the comparison between the different compounds, we also calculated the entry/exit barriers, similarly to the pure POPC systems using different methodologies (Table 1). From the data in Table 2, we calculated the correlation between the permeability coefficients and either the Δ*G*_entry_ or the Δ*G*_exit_ values. Similarly to the values for pure POPC (Table 1), we observed a strong negative correlation (−0.83) for Δ*G*_entry_ and a significantly smaller value for Δ*G*_exit_ (−0.28). With such a high anticorrelation between the membrane permeability values and the Δ*G*_entry_, we could argue that the estimation of this energy barrier will suffice to quickly gauge the membrane PAINS-like potential of a compound. It should be noted that the calculations required to estimate the entry barrier are only a modest fraction of the total needed to calculate the complete energy profile.

## Conclusion

Membrane PAINS are promiscuous compounds that can alter membrane physicochemical properties and perturb the function of transmembrane mechanosensitive proteins. To avoid the waste of time and resources in drug discovery companies due to this class of compounds, we have devised a computational protocol on the basis of umbrella sampling MD simulations to discriminate between compounds with differentiated membrane PAINS behavior. By coupling a molecular probe to this robust atomistic sampling scheme, we concluded that this method strongly depends on the specific environment sensed by the probe, especially at the membrane/water interface. To mitigate the impact of this heterogeneity, we changed the US reference position from the membrane center to the closest interacting monolayer P atoms. This approach resulted in a higher homogeneity between replicates, in particular, the description of the energetic barrier at the water/membrane interface. Additionally, in order to decrease the computational cost of such demanding simulations while retaining a high accuracy, we evaluated a reduction in the number of replicates, umbrellas, and simulation length. The final optimized scheme focused mainly on a reduced number of umbrellas, which significantly decreased the computational time spent without compromising the overall accuracy. This final optimized protocol was then applied to membrane systems in the presence of three compounds with different reported membrane PAINS behaviors: curcumin (membrane PAINS), resveratrol (mild membrane PAINS), and nothofagin (nonmembrane PAINS). The membrane permeability coefficients calculated using the inhomogeneous solubility-diffusion model for the different systems confirmed that resveratrol has mild membrane PAINS characteristics and that curcumin exhibits a concentration-dependent membrane PAINS behavior. However, our results indicate that nothofagin, which was previously identified as a nonmembrane PAINS compound,^16^ presents a significant membrane perturbation effect, suggesting a misclassification of this compound. The high accuracy and robustness of our protocol comes at a significant computational cost; therefore, it should also be used as a reference control for future developments aiming at faster protocols. Interestingly, we have observed a very high anticorrelation between the probe entry energy barrier (Δ*G*_entry_) and the membrane PAINS character. This can be an important step toward faster methods since this energy barrier can be calculated using just a fraction of our protocol. Overall, our effective approach emerges as a reference *in silico* method to identify and discriminate among membrane PAINS compounds.

## Data and Software Availability

The GROMACS package is a freely available software used to perform MD simulations and can be downloaded at https://manual.gromacs.org/documentation/2018.6/download.html. PyMOL v2.0 is also a free software for molecular visualization and the generation of high quality images. It can be downloaded from https://pymol.org/2. Gnuplot 5.4 is a portable command-line driven graphing utility used to generate plots, and it is freely available from http://www.gnuplot.info/download.html. Gimp 2.10.30 is a free and open source software image editor that can be downloaded from https://www.gimp.org/downloads/. Additionally, as Supporting Information, we provide the system starting configurations and topologies.

## Supporting Information Available

Time series of membrane total area, monolayer local thickness, and probe positions in the US scheme; membrane insertion distributions of all compounds studied; histogram distributions of the probe positions (pullx) for the different schemes; local thickness per umbrella from the 37U^M^ system; table with the positions and force constants used in the 22U^M^ system (PDF)
