# Supplementary material for: Optimization of an in Silico Protocol Using Probe Permeabilities to Identify Membrane Pan-Assay Interference Compounds
Source: J Chem Inf Model. 2022 Jun 13;62(12):3034–42. doi: 10.1021/acs.jcim.2c00372 (PMC9770580; doi:10.1021/acs.jcim.2c00372)
Supplement: Supplementary file 1 — ci2c00372_si_001.pdf [file ci2c00372_si_001.pdf]

Supporting Information: Optimization of an *in silico* protocol using probe permeabilities to identify membrane Pan-Assay Interference Compounds

Pedro R. Magalhães,[†] Pedro B. P. S. Reis,[†] Diogo Vila-Viçosa,[‡] Miguel Machuqueiro,[*,†] and Bruno L. Victor[*,†]

†*BioISI - Biosystems & Integrative Sciences Institute, Faculty of Sciences, University of Lisboa, Campo Grande, C8 bdg, 1749-016 Lisboa, Portugal*

‡*Kinetikos, 3030-199 Coimbra, Portugal*

E-mail: machuque@ciencias.ulisboa.pt; blvictor@ciencias.ulisboa.pt

Phone: +351-21-7500112; +351-21-7500000

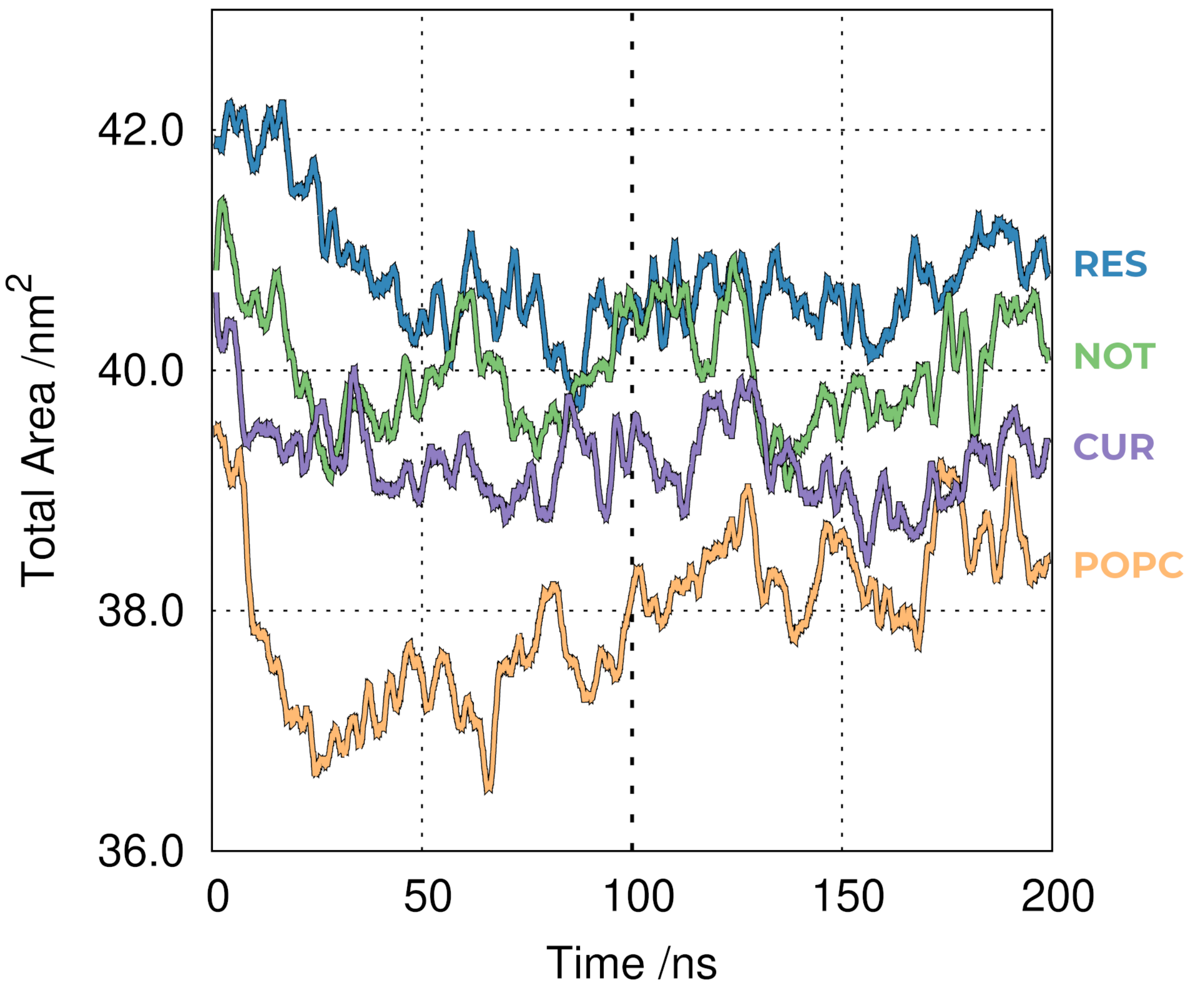

Figure S1: Total area of the membrane patch over time for the unbiased simulations of POPC in the absence and presence of different compounds – resveratrol (RES), nothofagin (NOT), and curcumin (CUR).

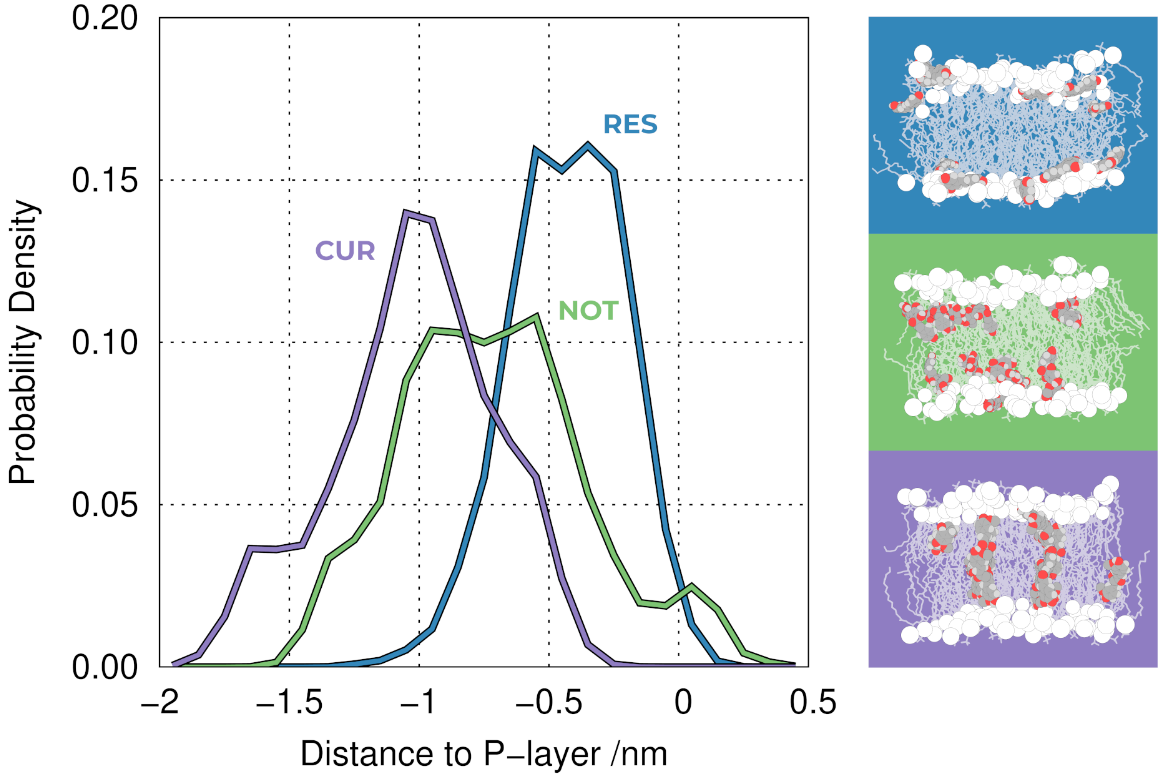

Figure S2: Membrane insertion distributions of resveratrol (RES), nothofagin (NOT), and curcumin (CUR) (left). The reference distance (zero) corresponds to the average position of the P-atoms (closest monolayer). Structural representations of the different compounds embedded in the membrane (RES, NOT and CUR, from top to bottom, on the right).

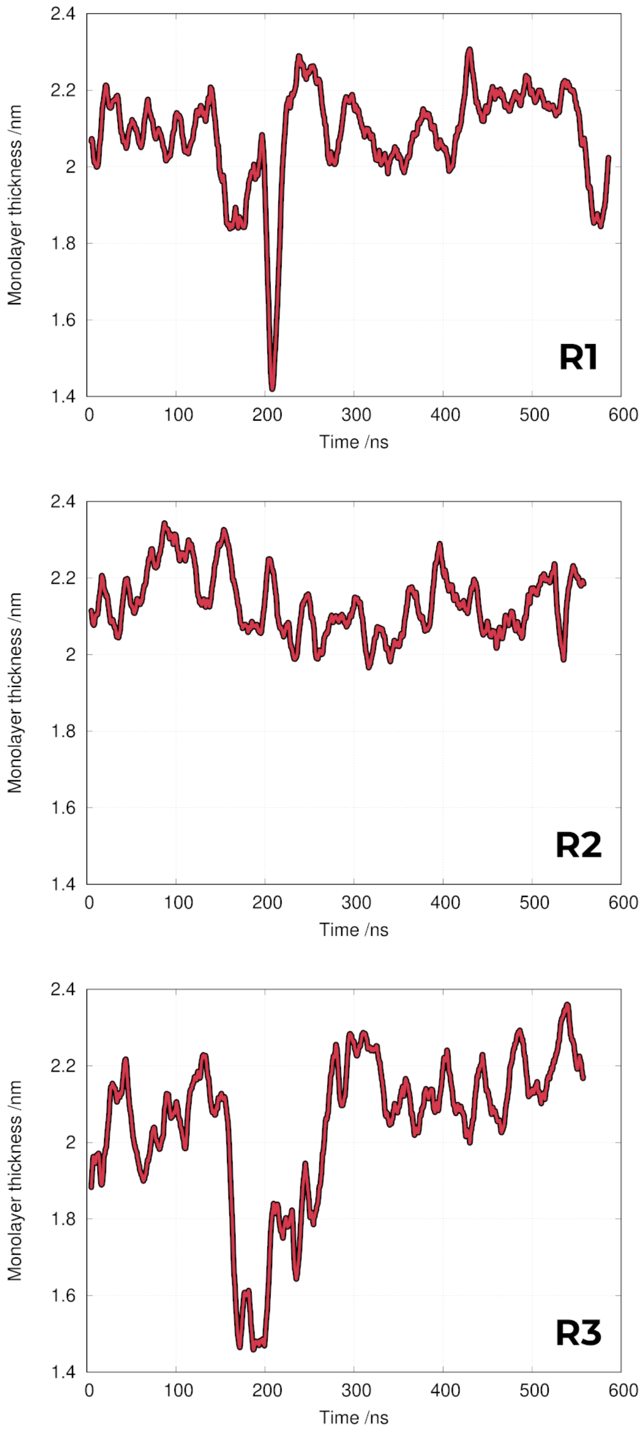

Figure S3: Local monolayer thickness over time for Umbrella 1.9 nm of the three replicates from system $37U^B$. This thickness was calculated using as reference all P-atoms within a 1.0 nm radius.

ISDM formalism

As described by Dickson and coworkers in ref. S1 and implemented in ref. S2, the position-dependent diffusion values $D(z)$ were calculated by:

$$D(z) = \frac{\mathrm{var}(z)^2}{\int_0^\infty C_{zz}(t)\mathrm{d}t} \tag{1}$$

where $C_{zz}(t) =< \delta z(0)\delta z(t) >$ is the autocorrelation function of the z-position of the LJ probe during the PMF window.

The position-dependent resistance values are then:

$$R(z) = \frac{\exp(\beta)\Delta G(z))}{D(z)} \tag{2}$$

where $\beta = 1/k_B T$ with k_{B} is Boltzmann's constant and T is the temperature. Integration of the $R(z)$ profile allows the calculation of the overall permeation coefficient P_{eff}

$$P_{\mathrm{eff}} = \frac{1}{\int_{-z_b}^{z_b} R(z)\mathrm{d}z} \tag{3}$$

where the integration extremes are in the water phase at either side of the membrane.

Below we show the diffusion (Figure S4A) and resistance (Figure S4B) profiles for the $22\mathrm{U}^{\mathrm{M}}$ system.

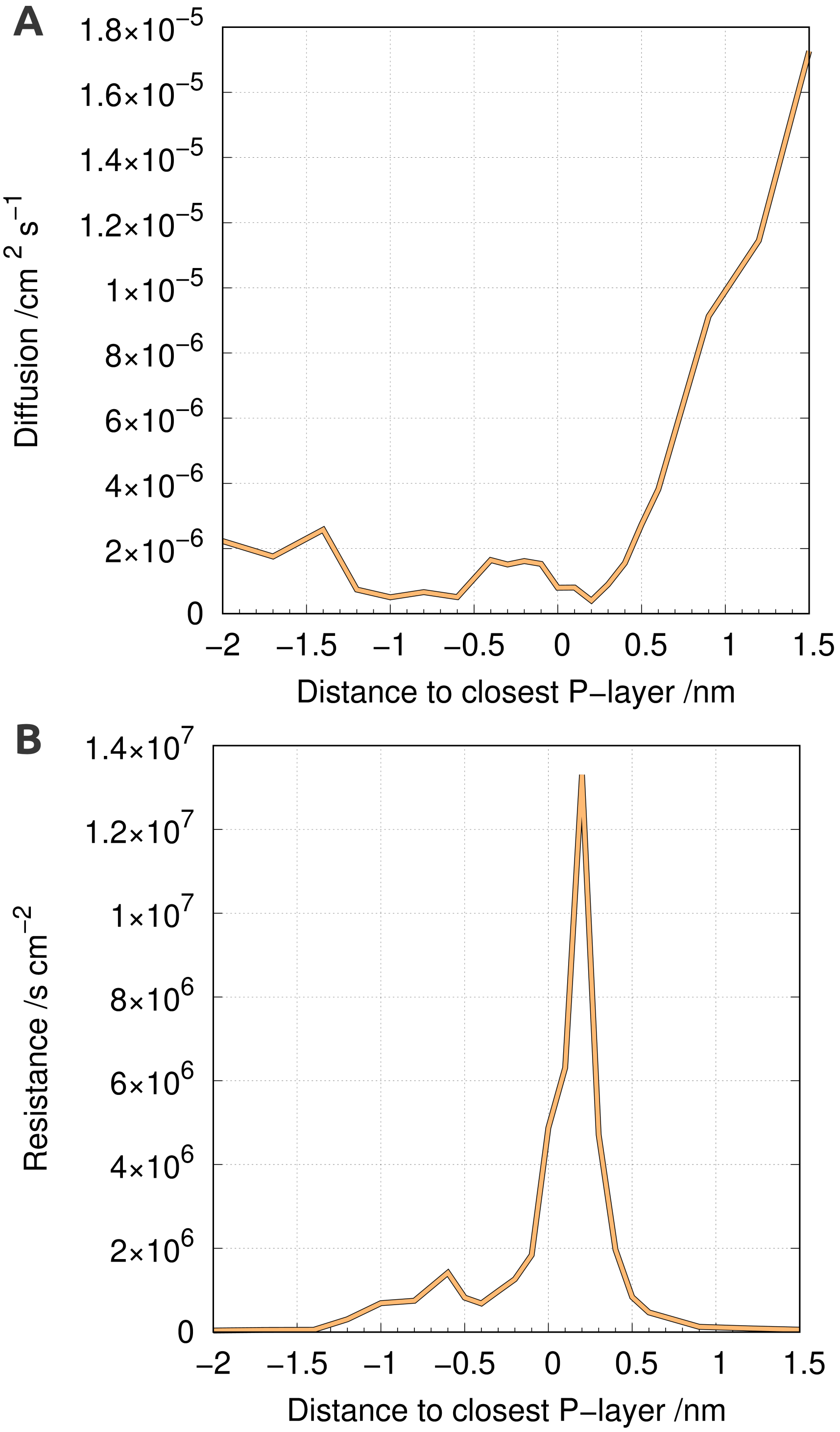

Figure S4: Diffusion (A) and resistance (B) profiles for the $22U^{M}$ system.

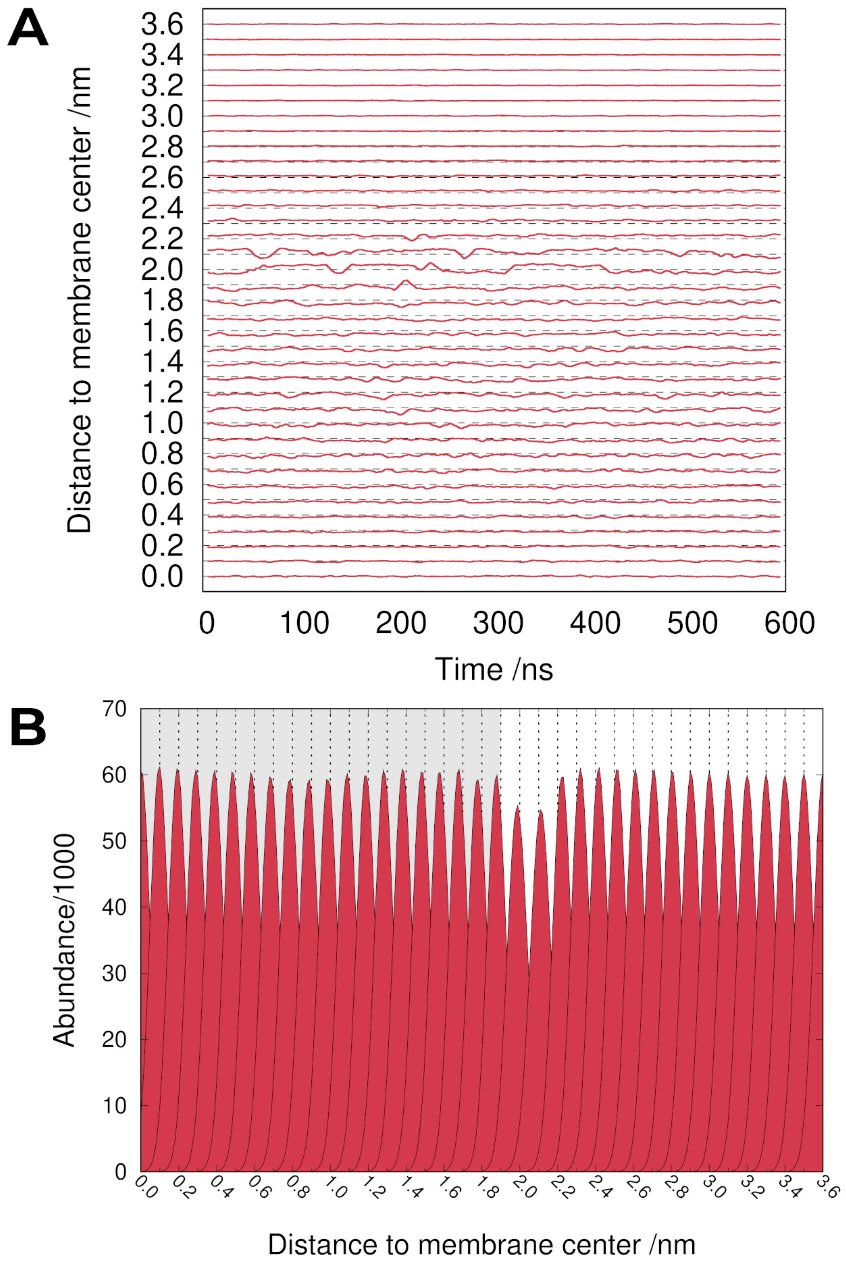

Figure S5: (A) Probe positions over time for the 37 umbrellas of replicate 1 in the pure POPC $37U^B$ system. (B) Histogram distributions for the same system, including all replicates. The grey-shaded area corresponds to the membrane region (using the POPC half-thickness - 1.89 nm - as delimiter).

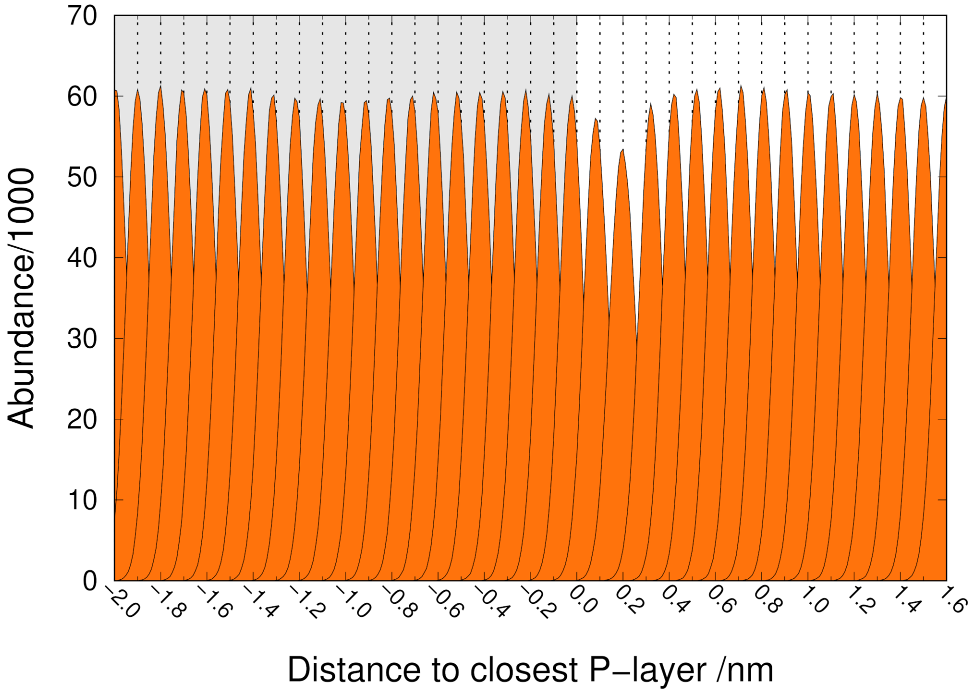

Figure S6: Histogram distributions for the 37 umbrellas of the the POPC $37U^{M}$ system, including all replicates. The grey-shaded area corresponds to the membrane region.

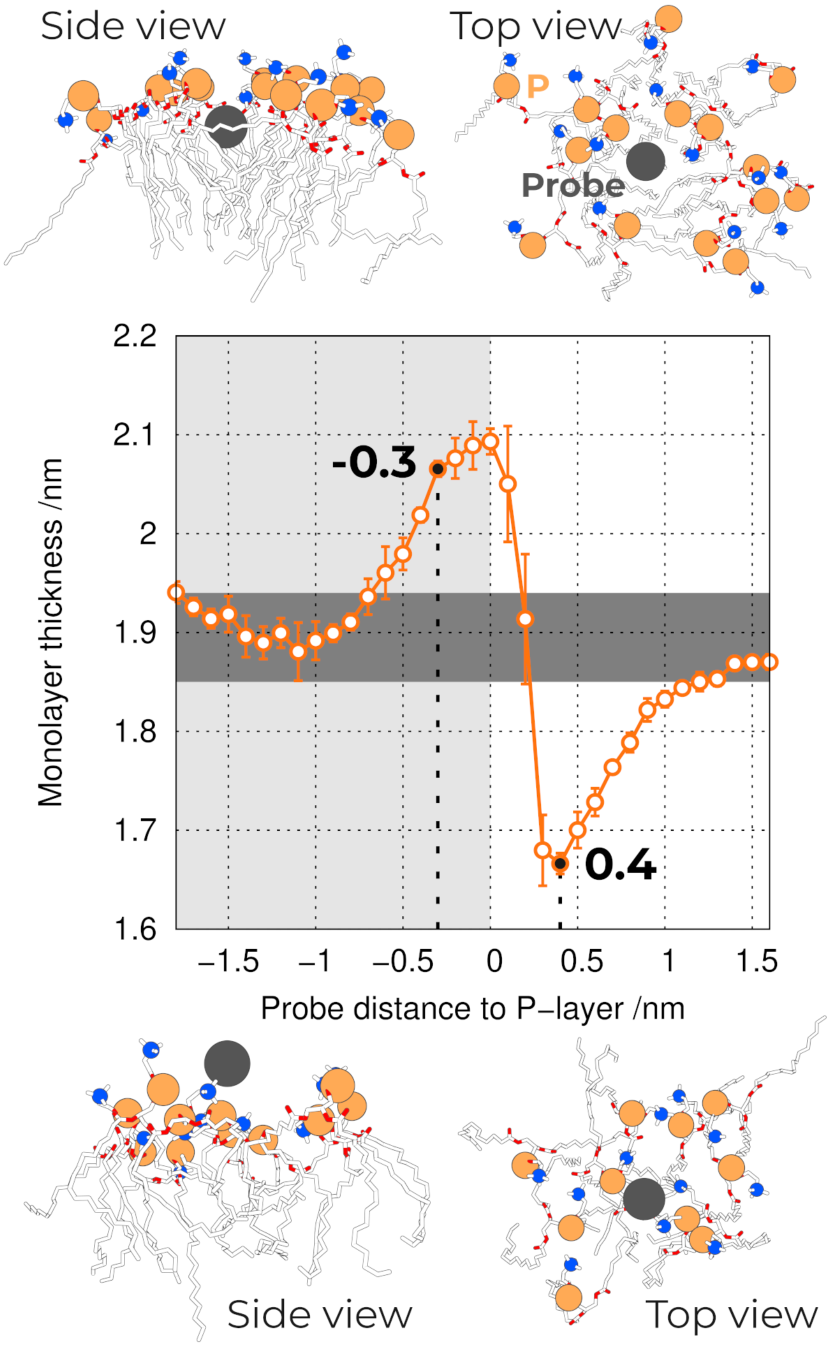

Figure S7: Local thickness per umbrella from the 37U^{M} system. The light grey region corresponds to the regions where the probe is inserted in the membrane. The dark grey region corresponds to the monolayer thickness values of unperturbed POPC (1.89±0.05 nm). Representative snapshots from two significantly deformed regions (-0.3 and +0.4 nm) are shown in side and top views.

Table S1: Position and corresponding force constants (K_f) used in the $22U^M$ system.

Umbrella position (nm)	K_f (kJ mol^{-1} nm^{-2})
−2.0	100
−1.7	100
−1.4	100
−1.2	500
−1.0	500
−0.8	500
−0.6	500
−0.5	1000
−0.4	2000
−0.3	2000
−0.2	2000
−0.1	2000
0.0	2000
0.1	2000
0.2	2000
0.3	2000
0.4	2000
0.5	1000
0.6	200
0.9	100
1.2	100
1.5	100

References

(S1) Dickson, C. J.; Hornak, V.; Pearlstein, R. A.; Duca, J. S. Structure–kinetic relationships of passive membrane permeation from multiscale modeling. *J. Am. Chem. Soc.* **2017**, *139*, 442–452.

(S2) Vila-Viçosa, D.; Victor, B. L.; Ramos, J.; Machado, D.; Viveiros, M.; Switala, J.; Loewen, P. C.; Leitão, R.; Martins, F.; Machuqueiro, M. Insights on the mechanism of action of INH-C_{10} as an antitubercular prodrug. *Mol. Pharm.* **2017**, *14*, 4597–4605.
